# Un cas de leishmaniose féline disséminée dans le sud de la France

**DOI:** 10.1051/parasite/2012191077

**Published:** 2012-02-15

**Authors:** E. Pocholle, E. Reyes-Gomez, A. Giacomo, P. Delaunay, L. Hasseine, P. Marty

**Affiliations:** 1 Université de Liège, boulevard de Colonster 20 4000 Liège (Sart Tilman) Belgique; 2 Université Paris-Est, École Nationale Vétérinaire d’Alfort, Unité d’Embryologie, d’Histologie et d’Anatomie Pathologique 94704 Maisons-Alfort France; 3 29, boulevard François Suarez 06340 La Trinité France; 4 Service de Parasitologie-Mycologie, CHU l’Archet, Université de Nice-Sophia Antipolis, Inserm U895 BP 3079 06202 Nice Cedex 3 France

**Keywords:** *Leishmania infantum*, chat, ulcère cutané, virus de l’immunodéficience féline, allopurinol, *Leishmania infantum*, cat, cutaneous ulcer, Feline Immunodeficiency Virus, allopurinol

## Abstract

Cet article rapporte un cas de leishmaniose féline disséminée chez un chat (*Felis catus*) de 14 ans, séropositif pour le FIV et vivant dans les Alpes-Maritimes (sud de la France). Le chat présente des papules érythémateuses ulcérées sur la face et l’encolure, et une lésion proliférative ulcérée sur l’oreille gauche. C’est l’examen histopathologique des lésions cutanées qui permet le diagnostic d’une leishmaniose disséminée, associée à un carcinome épidermoïde de l’oreille. 100 mg d’allopurinol administrés une fois par jour *per os* pendant quatre mois ont permis la rémission totale des lésions cutanées. Des prélèvements *post mortem* ont révélé la persistance du parasite dans l’organisme après six mois de traitement. Cet article discute de la sensibilité du chat à la leishmaniose et de son rôle potentiel de réservoir.

## Abstract (en)

We report a case of disseminated feline leishmaniasis in a FIV-seropositive 14-year-old male cat (*Felis catus*) living in the Alpes-Maritimes (south of France). The cat presented with erythematous ulcerated papules on the head and withers, and with an ulcerated proliferative lesion on the left pinnae. The condition was diagnosed, along with a squamous cell carcinoma of the pinnae, after histopathological examination of the cutaneous lesions. Total remission of the cutaneous lesions was obtained after oral administration of 100 mg of allopurinol once a day for four months. Necropsic samples revealed that the parasite was still present in the organism after six months of treatment. This case discusses of the cat sensibility to the leishmaniasis pathology and of his potential ability of being a reservoir host.

La leishmaniose, maladie endémique dans le sud de la France, est bien étudiée dans les espèces canine et humaine, mais le rôle du chat domestique (Felis catus) reste un sujet de controverse. En effet, on ignore s’il est un hôte accidentel, usuel ou un porteur asymptomatique. C’est ce que l’on propose de discuter dans cet article.

## Matériel et Méthodes

Pilou” est un chat européen mâle castré de 14 ans, de robe rousse et blanche, et aux oreilles blanches. L’animal vit essentiellement à l’extérieur près de Saint-André-de-la-Roche, un important foyer de leishmaniose canine, et n’a jamais voyagé. En décembre 2005, le chat est présenté pour une pododermatite récurrente. Le prurit est marqué au niveau des doigts qui présentent des lésions érythémateuses et exsudatives. Une bactériologie par écouvillonnage des lésions cutanées ne rapporte que quelques staphylocoques non pathogènes. Malgré cela, les traitements antibiotiques restent inefficaces. Un examen sérologique effectué à cause de la récurrence de la maladie (Speed^®^ Duo FeLV/FIV, Bio Veto Test, Virbac, 83500 La Seyne-Sur-Mer, France) révèle une positivité vis-à-vis du virus de l’immunodéficience féline (FIV). Une chimiothérapie à base d’une série d’interféron oméga (Virbagen Omega 10 ME^®^, Virbac) permet la disparition du prurit et des signes cliniques pendant trois ans.

**Figure 1.**
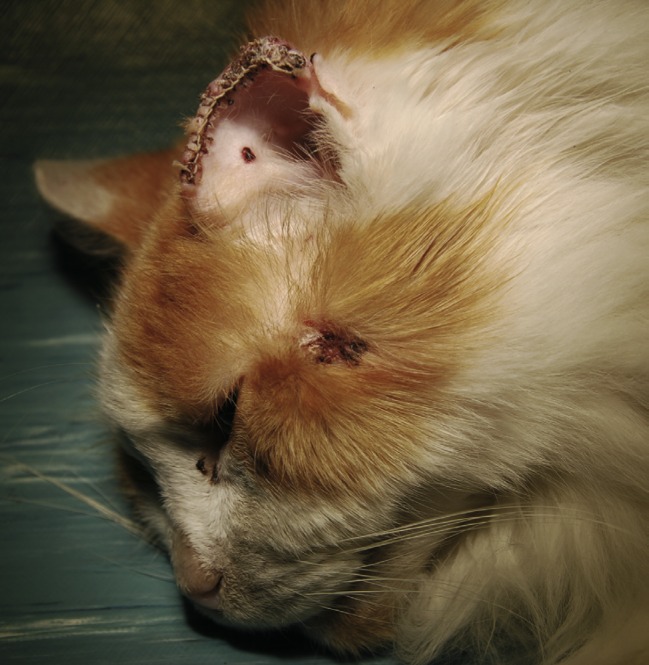
Chat de l’étude après la résection du pavillon auriculaire. Noter la lésion sur la joue gauche.

À l’examen clinique, en décembre 2008, le chat pèse 4 kg et présente une perte de poids de 20 % avec un état d’embonpoint en dessous de la normale. Son appétit est diminué et il est légèrement abattu. Il a trois lésions cutanées : une sur la tête, environ 2 cm en arrière de l’oeil gauche, une autre à la base de la face supérieure de l’oreille gauche et une dernière dans la région interscapulaire. Toutes ont un aspect de papule érythémateuse ulcéreuse et sanguinolente d’environ 1 cm de long sur quelques millimètres de large. Elles sont circonscrites et recouvertes par quelques squames et de petites croûtes (Figure 1). Sur le pavillon auriculaire gauche, on trouve une quatrième lésion étendue ressemblant à un ulcère prolifératif, localisée sur toute l’extrémité supérieure et dépigmentée de l’oreille et s’étendant progressivement à la partie pigmentée. Les lésions sont apparues en trois à quatre semaines environ.

Les lésions ulcératives d’un pavillon auriculaire dépigmenté chez un chat vivant à l’extérieur étant très fortement suspectes d’un carcinome épidermoïde, on procède à la résection du pavillon pour le soumettre à un examen histopathologique. Les lésions sur le garrot et à la base de l’oreille gauche, présentant suffisamment de peau pour être refermées, sont également biopsiées et analysées. Dans l’attente des résultats du laboratoire, le chat reçoit une injection sous-cutanée de 8 mg/kg PV de céfovécine (80 mg/ml – Convenia^®^, Pfizer A.H.) pour traiter les plaies cutanées et une nouvelle série d’interféron oméga.

## Résultats

### Examen anatomo-pathologique

Lésion auriculaire gauche : prolifération à point de départ épidermique, formée de cordons anastomosés à différenciation malpighienne, séparés par un stroma fibrobastique, avec un fort degré d’atypie et présence de mitoses anormales : carcinome épidermoïde invasif.

Lésions du garrot et de la base de l’oreille gauche : infiltration inflammatoire péri-vasculaire du derme superficiel s’étendant le long des annexes et masquée sous l’ulcération par une réaction fibroblastique sévère ; hyperkératose parakératosique associée à des croûtes séro-cellulaires renfermant de nombreuses colonies bactériennes de type cocci sur les marges de l’ulcère ; nombreux macrophages renfermant dans leur cytoplasme des éléments basophiles ovoïdes ou en virgules, d’environ 1 μm (corps de Leishman-Donovan) : forte suspicion de leishmaniose.

Pour confirmer le diagnostic de leishmaniose, le chat est anesthésié dix jours plus tard en vue de l’exérèse de la lésion de la joue gauche et d’une analyse sérologique spécifique.

### Examen parasitologique complet sur la biopsie de peau

Examen microscopique : nombreux amastigotes de *Leishmania* sp. (techniques d’apposition avec coloration au May Grünwald Giemsa (Lima *et al.*, 1997)).

Mise en culture sur milieu Nicolle-Novy-McNeal et de Schneider (Lima *et al.*, 1997) : promastigotes de *Leishmania* sp. et isolement de la souche MFEL/FR/09/LPN 326 dont l’identification isoenzymatique au Centre National de Références des Leishmanioses de Montpellier a montré qu’il s’agit du zymodème MON-1 de *Leishmania infantum*, zymodème classique chez le chien et dans la plupart des cas viscéraux humains du sud de la France.

Confirmation du résultat anapathologique : présence de *Leishmania* dans la peau.

### Tests sérologiques et hématologiques

Western Blot : positif avec présence des bandes 14, 18, 21 et 31 kDa typiques de la leishmaniose viscérale patente humaine (Marty *et al.*, 1994 ; Suffia *et al.*, 1995).

Examen microscopique après leuco-cytocentrifugation du sang et coloration de May Grünwald Giemsa : négatif.

Culture du sang sur milieu de Schneider : positive avec présence de promastigotes.

PCR en temps réel ciblant l’ADN du kinétoplaste des leishmanies (Mary et al., 2004) : parasitémie à 26 parasites par millilitre.

### Évolution

La présence de leishmanies dans les plaies cutanées et dans le sang confirme le diagnostic de leishmaniose féline disséminée. On prescrit alors l’allopurinol (Zyloric^®^, GlaxoSmithKline) à 100 mg par jour en une seule prise orale que l’on maintient jusqu’à disparition des symptômes. En avril 2009, on observe une amélioration clinique nette : les plaies cutanées ont cicatrisé, le prurit a disparu et aucune rechute du carcinome n’est observée. Le chat a repris du poids et est très vif. Du sang est prélevé : les cultures sont négatives, mais la PCR en temps réel rapporte encore une parasitémie faible d’environ 11 parasites par millilitre.

Le chat décède le 18 juillet 2009 à la suite d’un accident de la voie publique. Une autopsie pratiquée deux jours après révèle des réserves adipeuses bien développées confirmant l’amélioration clinique. Une culture sur milieu de Schneider est réalisée pour la rate et le foie : seule la rate présente des promastigotes de *Leishmania* sp. La PCR en temps réel montre la présence d’ADN de leishmanies dans les deux organes.

## Discussion

Dans les Alpes-Maritimes, foyer endémique de *L. infantum*, seulement deux cas de leishmaniose féline disséminée ont été décrits. Les deux chats présentaient des lésions cutanées avec dispersion de *L. infantum* dans la moelle osseuse ou dans la rate et les ganglions (Ozon *et al.*, 1998 ; Grevot *et al.*, 2005). L’un d’entre eux était séropositif pour le FIV et le virus leucémogène félin (FeLV), mais restait asymptomatique avec un bon état général. Le cas du chat Pilou ne fait donc pas exception : les examens parasitologiques montrent la persistance de *Leishmania* dans le sang après quatre mois de traitement, et l’autopsie confirme la présence du parasite dans la rate et le foie après six mois de traitement. Il est donc probable que, comme dans l’espèce canine, la leishmaniose chez le chat ne puisse être scindée en maladie cutanée et maladie viscérale, mais prenne plutôt un aspect systémique. Cela démontre aussi que, cliniquement guéri et toujours sous traitement, l’animal est encore porteur de leishmanies circulantes et devient donc un porteur asymptomatique. Les agents “immunosuppresseurs” comme le FIV et le FelV ou le stress pourraient contribuer au développement de la maladie, mais aucune corrélation n’a été prouvée à ce jour (Maia *et al.*, 2010 ; Martin-Sanchez *et al.*, 2007 ; Hervas *et al.*, 1999).

Les signes cliniques chez le chat sont proches de ceux du chien et sont généralement provoqués par *L. infantum* MON-1. Ils s’expriment par une alopécie et des lésions nodulaires ou parfois papuleuses, ulcéreuses ou croûteuses au niveau du nez, des lèvres, des oreilles et des paupières. On observe aussi une dissémination sanguine préférentielle au niveau de la rate, du foie, de la moelle osseuse et des ganglions lymphatiques (Bourdoiseau, 2011 ; Trainor *et al.*, 2010 ; Marty *et al.*, 2007, Grevot *et al.*, 2005, Poli *et al.*, 2002 ; Ozon *et al.*, 1998). Au niveau microscopique, les lésions cutanées ont généralement l’aspect d’une inflammation diffuse granulomateuse du derme avec des macrophages contenant des amastigotes de *Leishmania* associés à de nombreux neutrophiles, lymphocytes, plasmocytes et mastocytes (Navarro *et al.*, 2010). L’épiderme peut être hyperkératosique, hyperplasique et est souvent ulcéré (Trainor *et al.*, 2010). Les lésions viscérales montrent une hypoplasie de la rate avec de nombreux macrophages très infestés en zone marginale, dans les follicules lymphoïdes ou autour des artérioles. Dans le foie, l’infiltrat de macrophages fortement infestés se localise dans les espaces portes et en petits foyers entre les hépatocytes (Navarro *et al.*, 2010).

Une étude sérologique réalisée sur des chats asymptomatiques de la région de Nice révèle 12 % de chats séropositifs, soit un résultat similaire à la prévalence canine (12,4 %) (Marty *et al.*, 2007 ; Grevot *et al.*, 2005). Les chats peuvent donc être des porteurs asymptomatiques (Maia *et al.*, 2008 ; Maia *et al.*, 2010). Par ailleurs, Maroli *et al.* ont montré en 2007 qu’un chat chroniquement infecté peut transmettre *Leishmania* sp. à son vecteur principal en Italie (*Phlebotomus perniciosus*). L’espèce féline pourrait donc jouer un rôle de réservoir de *L. infantum* car il y a un haut pourcentage de chats parasitémiques et une transmission possible du parasite à son vecteur. La leishmaniose doit donc être incluse dans le diagnostic différentiel des lésions cutanées ulcératives si le chat vit ou a voyagé dans une région endémique.
